# Supplementary material for: Integrating genomic structural equation modeling and experimental validation to unravel the genetic basis of male genital lichen sclerosus
Source: Front Immunol. 2026 Jul 16;17:1857945. doi: 10.3389/fimmu.2026.1857945 (PMC13435250; doi:10.3389/fimmu.2026.1857945)
Supplement: Supplementary file 6 [file SupplementaryFile5.zip › result/fuma_PSYCH_factor_sCCA2_focus.chr16.chr16.20150571.22448904.pop1.pdf]

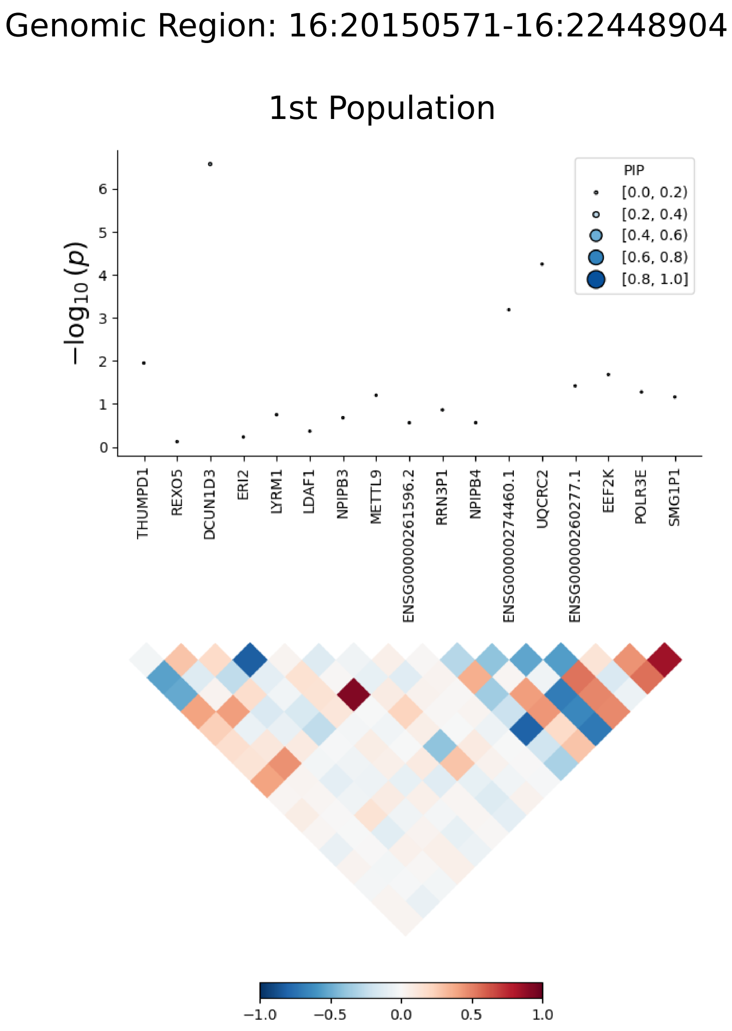
Genomic Region: 16:20150571-16:22448904
1st Population
$-\log_{10}(p)$
PIP
[0.0, 0.2)
[0.2, 0.4)
[0.4, 0.6)
[0.6, 0.8)
[0.8, 1.0]
THUMPD1
REXO5
DCUN1D3
ERI2
LYRM1
LDAF1
NPIPB3
METTL9
ENSG00000261596.2
RRN3P1
NPIPB4
ENSG00000274460.1
UQCRC2
ENSG00000260277.1
EEF2K
POLR3E
SMG1P1
−1.0
−0.5
0.0
0.5
1.0
